# Electron Signatures of Reconnection in a Global eVlasiator Simulation

**DOI:** 10.1029/2022GL098329

**Published:** 2022-07-20

**Authors:** M. Alho, M. Battarbee, Y. Pfau‐Kempf, Yu. V. Khotyaintsev, R. Nakamura, G. Cozzani, U. Ganse, L. Turc, A. Johlander, K. Horaites, V. Tarvus, H. Zhou, M. Grandin, M. Dubart, K. Papadakis, J. Suni, H. George, M. Bussov, M. Palmroth

**Affiliations:** ^1^ Department of Physics University of Helsinki Helsinki Finland; ^2^ Swedish Institute of Space Physics Uppsala Sweden; ^3^ Space Research Institute Austrian Academy of Sciences Graz Austria; ^4^ Finnish Meteorological Institute Helsinki Finland

**Keywords:** kinetic simulation, reconnection, electron distribution function, magnetosphere, vlasov equation, electric field

## Abstract

Geospace plasma simulations have progressed toward more realistic descriptions of the solar wind–magnetosphere interaction from magnetohydrodynamic to hybrid ion‐kinetic, such as the state‐of‐the‐art Vlasiator model. Despite computational advances, electron scales have been out of reach in a global setting. eVlasiator, a novel Vlasiator submodule, shows for the first time how electromagnetic fields driven by global hybrid‐ion kinetics influence electrons, resulting in kinetic signatures. We analyze simulated electron distributions associated with reconnection sites and compare them with Magnetospheric Multiscale (MMS) spacecraft observations. Comparison with MMS shows that key electron features, such as reconnection inflows, heated outflows, flat‐top distributions, and bidirectional streaming, are in remarkable agreement. Thus, we show that many reconnection‐related features can be reproduced despite strongly truncated electron physics and an ion‐scale spatial resolution. Ion‐scale dynamics and ion‐driven magnetic fields are shown to be significantly responsible for the environment that produces electron dynamics observed by spacecraft in near‐Earth plasmas.

## Key Points

- We present a survey of kinetic electron distribution functions in a global Vlasov model of the Earth's magnetosphere
- Ion‐scale background fields yield electron distributions consistent with Magnetospheric Multiscale observations
- Parallel electric fields are observed at the magnetopause, coincident with a flat‐top electron distribution

## 1 Introduction

Understanding of the near‐Earth space has, in recent decades, evolved from describing magnetized fluids to ion‐scale physics and even smaller spatial domains controlled by electron‐scale physics. Electron‐scale physics is especially important in understanding magnetic reconnection, the main process of energy conversion in the magnetosphere (Yamada et al., 2010). Previously observed electron signatures of reconnection include the *Cluster* observations described by Asano et al. (2006), while other observations of electron features in the magnetosphere by Nakamura et al. (2008) present field‐aligned currents with parallel electron heating in an ion‐scale current sheet. Electron velocity distribution functions (VDFs) can be very complex and deviate from Maxwellian and bi‐Maxwellian distributions in collisionless plasmas (Graham et al., 2021; Shuster et al., 2021).

In particular, the *Magnetospheric Multiscale* (MMS) mission (Burch et al., 2016) has recently provided for new high‐cadence, high‐resolution observations (Pollock et al., 2016) of electron VDFs in and around the Earth's magnetosphere. Reconnection processes and electron VDFs have been intensively studied with MMS in different regions, such as the magnetopause, where Khotyaintsev et al. (2016) and Wang et al. (2019) show dayside reconnection inflows and outflows, potentially in reach of our simulation. Chen, Hesse, Wang, Gershman, et al. (2016) and Li et al. (2020) detail perpendicular crescent observations and Webster et al. (2018) survey other electron diffusion region (EDR) observations, which we might not resolve. In the magnetotail, Nakamura et al. (2016) and Wang et al. (2018) show thin electron current sheets at the plasma sheet boundary layer (PSBL) and Varsani et al. (2017) and Wellenzohn et al. (2021) investigate electron reconnection signatures in terms of velocity dispersion. While velocity dispersion may not be a suitable target for our simulation, the plasma sheet boundary layer was previously noted as an interesting target (Battarbee et al., 2021). Chen et al. (2020) discuss lower hybrid waves and Grigorenko et al. (2020) describe whistler‐related electron observations at dipolarization fronts, falling outside the scope of our simulations. Tail reconnection sites have been studied closely at the EDR regions with MMS by Torbert et al. (2018) and Chen et al. (2019), but as with the dayside EDRs, we do not reach these length scales. Out of these observations, we hope to model electron behavior related to reconnection sites without trying to inspect the EDR due to computational constraints.

Specifically on the dayside, we look at the results of Khotyaintsev et al. (2020), who describe electron observations at the magnetopause in relation to asymmetric reconnection. Detailed VDFs are given along with observations of electrostatic wave activity proposed to explain features of the VDFs. In the magnetotail, we focus on the results of Nakamura et al. (2016), who detail small‐scale, field‐aligned current sheets and their associated electron distributions, showing parallel beaming. However, these in situ observations allow only local snapshots of the controlling physics without the possibility to gain information on the adjacent processes that may influence distribution functions.

With modeling, we can provide a global context to in situ spacecraft observations. Advances in computational capabilities have enabled the development of increasingly complex and descriptive plasma models from magnetohydrodynamic (MHD) simulations (Janhunen et al., 2012; Tóth et al., 2012; Zhang et al., 2019) and test Vlasov (Palmroth et al., 2013) to ion‐kinetic hybrid models (Karimabadi et al., 2014; Omelchenko et al., 2021; Valentini et al., 2007), and to fully kinetic simulations of electron microphysics (Daughton et al., 2011; Pezzi et al., 2019; Schmitz & Grauer, 2006), including simulations of EDRs (Chen, Hesse, Wang, Bessho, & Daughton, 2016; Hesse et al., 2016; Hoshino et al., 2001; Wilson et al., 2016). Due to computational restrictions, simulations depicting electron VDFs are, while not as local as the spacecraft measurements, spatially restricted, and the self‐consistent electron‐kinetic regime in global geospace plasma simulations has remained out of reach. Progress has been made in terms of embedding full‐kinetic particle‐in‐cell (PIC) regions into MHD simulations with MHD‐EPIC (Chen et al., 2017; Daldorff et al., 2014) albeit for scaled ion inertial lengths and increased electron masses (Tóth et al., 2017). Recently, adaptive kinetic regions have been introduced in MHD‐AEPIC (Shou et al., 2021) for a local setting. Alternative methods for including electrons in hybrid simulations have been previously brought forward by Lin and Chen (2001) in terms of kinetic corrections to a fluid electron model and by Tronci and Camporeale (2015), describing a variational generation of a kinetic neutral Vlasov theory.

Working toward globally described electron VDFs, Battarbee et al. (2021) presented the technical details and local‐scale validation of a new electron propagation scheme, eVlasiator, which uses existing Vlasiator ion‐kinetic global simulations as a starting point for global electron simulations. The eVlasiator method propagates electron distribution functions in constant background fields, describing the VDF evolution with the Vlasov equation and including electron plasma oscillations. The global plasma dynamics drive reconnection and the electron‐scale physics, and the objective of eVlasiator is to understand the local electron VDFs within this global context.

In this work, we apply eVlasiator (Battarbee et al., 2021) to model the global magnetosphere for the first time. This approach probes which parts of the electron VDFs can be understood in terms of global ion‐scale physics and which parts—not modeled by eVlasiator—stem from electron‐scale kinetic effects. We carry out a two‐dimensional (2D) simulation featuring a three‐dimensional velocity space (3V) for electrons and compare electron VDFs close to dayside and nightside reconnection sites with previously published MMS observations by Nakamura et al. (2016) and Khotyaintsev et al. (2020), placing these observational results within a larger spatial context.

## 2 Model and Methods

### 2.1 Vlasiator and eVlasiator

Vlasiator (Palmroth et al., 2018; von Alfthan et al., 2014) is a global hybrid‐Vlasov simulation describing the near‐Earth space within the ion‐kinetic regime in 3 spatial dimensions. Each spatial cell includes a 3D ion velocity space. Vlasiator solves the ion Vlasov‐Maxwell system with the Darwin and quasineutral approximations and describes electrons as a charge‐neutralizing, massless fluid. eVlasiator (Battarbee et al., 2021) is a specialized solver building on the Vlasiator platform to model kinetic electrons with Vlasov methods at ion scales.

In eVlasiator, a Maxwellian electron population is initialized using the magnetic field and the moments of the proton distribution functions of a Vlasiator simulation as follows:

1. Equal number density to protons: *n*_e_ = *n*_p_,
2. Bulk velocity from ${\vec{V}}_{e}={\vec{V}}_{p}-\vec{J}/\left(n_{e}e\right)$, where $\vec{J}=\nabla \times \vec{B}/\mu_{0}$, *μ*_0_ is the vacuum permeability, $\vec{B}$ is the magnetic flux density, and ${\vec{V}}_{p}$ is the proton bulk velocity,
3. Temperature using an empirical proton‐electron temperature ratio *T*_p_/*T*_e_ = 4 (Artemyev et al., 2011; Wang et al., 2012).

The eVlasiator simulation grid is kept congruent with the previous Vlasiator solution to avoid resampling.

The initial state is propagated in time using the Vlasov equation and the Vlasiator methods with few changes. The proton moments are kept constant as the electron simulation time scale is much shorter than the characteristic time scales of proton dynamics. The magnetic field is also kept constant as the time scales of magnetic fluctuations at the available spatial scales (with spatial grid cell size Δ*x* = 300 km on the order of or below the ion inertial length in the tail) are much longer than the electron simulation timescale. Only the electron distributions are propagated according to the Vlasov equation, which is solved in a leapfrog fashion, alternating spatial translations and velocity space accelerations. In addition to magnetic force and convective and electron pressure gradient electric fields, the electrons experience electric fields ${\vec{E}}_{J_{e}}$ as a result of their oscillations, solved in tandem with the electron bulk velocity ${\vec{V}}_{e}$ (Battarbee et al., 2021):

(1) $$\delta {\vec{E}}_{J_{e}}=\delta t\;c^{2}\left(\nabla \times \vec{B}+\mu_{0}e\left(n_{e}{\vec{V}}_{e}-n_{p}{\vec{V}}_{p}\right)\right)$$

(2) $$\delta {\vec{V}}_{e}=\delta t\;\frac{e}{m_{e}}{\vec{E}}_{J_{e}}$$

in a coupled Runge‐Kutta 4 scheme, with ${\vec{E}}_{J_{e},t_{0}}=0$ for the initial eVlasiator step, *δt* the Runge‐Kutta timestep, and *c* the speed of light. See Battarbee et al. (2021) for a full description of the model.

#### 2.1.1 Resonant Case Handling

In Battarbee et al. (2021), a concern is raised for the coupled electron acceleration–electric field solver when the electron plasma oscillations are near resonance with electron cyclotron motion, that is, *ω*_pe_ ≈ *ω*_ce_, and increased electron substepping is suggested as a solution. This implies the electron Alfvén velocity approaching the speed of light. Still, the model does not include relativistic effects. To safeguard against potential numerical instabilities in this regime, we introduce an additional factor *k* to the number of electron oscillation solver substeps *N*, so that the new number of substeps is *N*′ = *kN* with

(3) $$k=\max\left(1,\min\left(100,|\log\left(\left|\omega_{\text{ce}}/\omega_{\text{pe}}\right|\right){|}^{-1}\right)\right).$$

This increases the number of substeps near the resonance point (up to the clamping factor of 100), which is found to improve solver stability in single‐cell tests without requiring excessive substeps in nonresonant regions. The number of substeps is not allowed to decrease with this method with a constraint of *k* > = 1 to avoid compromising solver stability.

### 2.2 Simulation Setup

**Figure 1:**
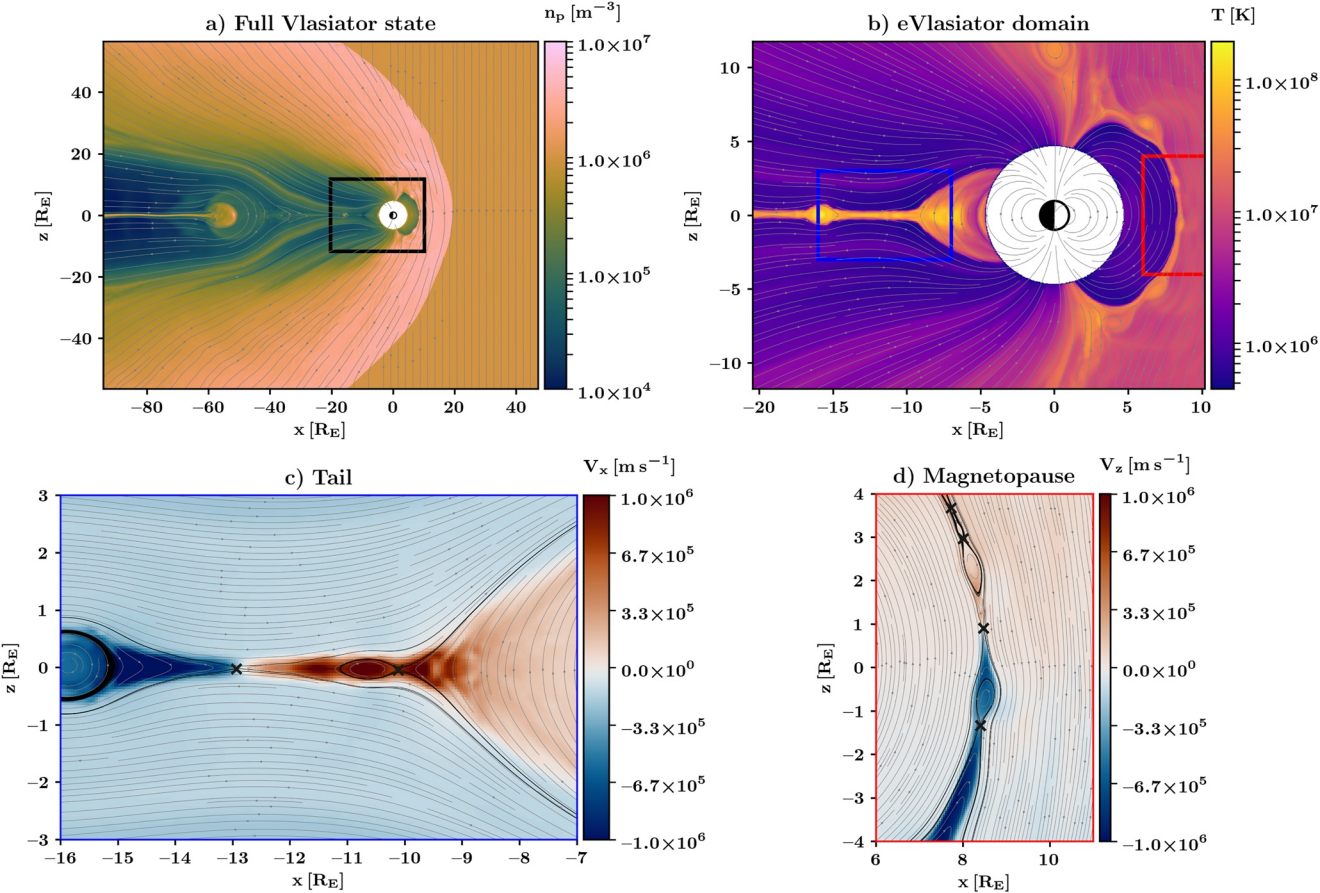
Overview of the input data set. (a) The full domain of the underlying simulation, showing proton density (color scale), magnetic field lines (gray), and the outline of the eVlasiator domain (b) in black. (b) The domain used for eVlasiator, showing proton temperature (color scale), magnetic field lines (gray), and outlines of detail panels c (blue) and d (red). Details of the input state in the tail current sheet (c) and dayside magnetopause (d), showing proton *V*_*x*_ and *V*_*z*_, respectively, as reconnection proxies, magnetic field lines in gray, separatrices as black lines, and X‐points as black crosses.

We use as input a snapshot of a noon‐midnight meridional plane simulation in Geocentric Solar Ecliptic (GSE) coordinates, and the same coordinate system is used in this work. The Earth's dipole is modeled by a line dipole parallel to the Z_GSE_ axis. The Vlasiator run used for initialization has been described by Hoilijoki et al. (2017) and studied in further detail by Palmroth et al. (2017) and Runov et al. (2021). The run produces variable‐rate dayside reconnection regardless of the time‐stationary solar wind, influenced by dayside flux transfer events (FTEs) and magnetosheath fluctuations (Hoilijoki et al., 2017). The tail reconnection includes considerable dynamics, influenced both by dayside‐driven global dynamics and bursty bulk flows (Juusola et al., 2018; Palmroth et al., 2017). Figure 1 shows the Vlasiator simulation used in initialization with Figure 1a showing the full domain, Figure 1b the eVlasiator domain, and Figures 1c and 1d show zoom‐in views depicting the magnetotail and dayside regions of further interest, respectively.

We take the simulation snapshot at 1925s, a time of considerable tail reconnection activity, with several X‐lines in the tail current sheet, and a primary reconnection site at around *X* ≈ −12.5 R_E_. There is a secondary earthward X‐line close to the transition region at *X* ≈ −10 R_E_ (Palmroth et al., 2017). Tailward of the primary reconnection site, we see a plasmoid at *X* ≈ −16 R_E_. Several FTEs are present on the dayside at this time (Hoilijoki et al., 2017). The chosen snapshot time captures interesting dynamics, comparable to observations by Nakamura et al. (2016); Varsani et al. (2017); and Khotyaintsev et al. (2020).

For the eVlasiator simulation, we choose a configuration with a modified electron mass *m*_e,sim_ = 10 *m*_e_ (so that *m*_e,sim_ ≈ 183.6^−1^ *m*_p_, proton mass) to reduce velocity space extents, memory usage, and runtime to manageable amounts. The electron velocity space covers ±42,000 km s^−1^ in each velocity dimension with a resolution of Δ*v* = 210 km s^−1^.

As in Battarbee et al. (2021), boundary conditions are static based on the initial conditions given by the Vlasiator input. The Vlasiator sparse velocity grid (von Alfthan et al., 2014) uses a sparsity threshold below which VDF contributions for specific velocity cells are ignored, but we enforce mass conservation by rescaling the VDFs by the ignored amount. This may artificially cool the distributions but the effect is negligible. This results in approximately 292 × 10^9^ velocity cells to be propagated during each timestep. We run the eVlasiator simulation for 1 s, a total of 144,313 timesteps at Δ*t* ≈ 6.9 μs; for reference, the electron cyclotron period *τ*_c,e_ varies in the range of 20–400 ms for the simulated *m*_e_. This allows the electron VDFs to evolve toward a quasi‐steady state, while the ion‐scale dynamics remain small enough to neglect.

## 3 Results

### 3.1 Analysis of the Simulation

**Figure 2:**
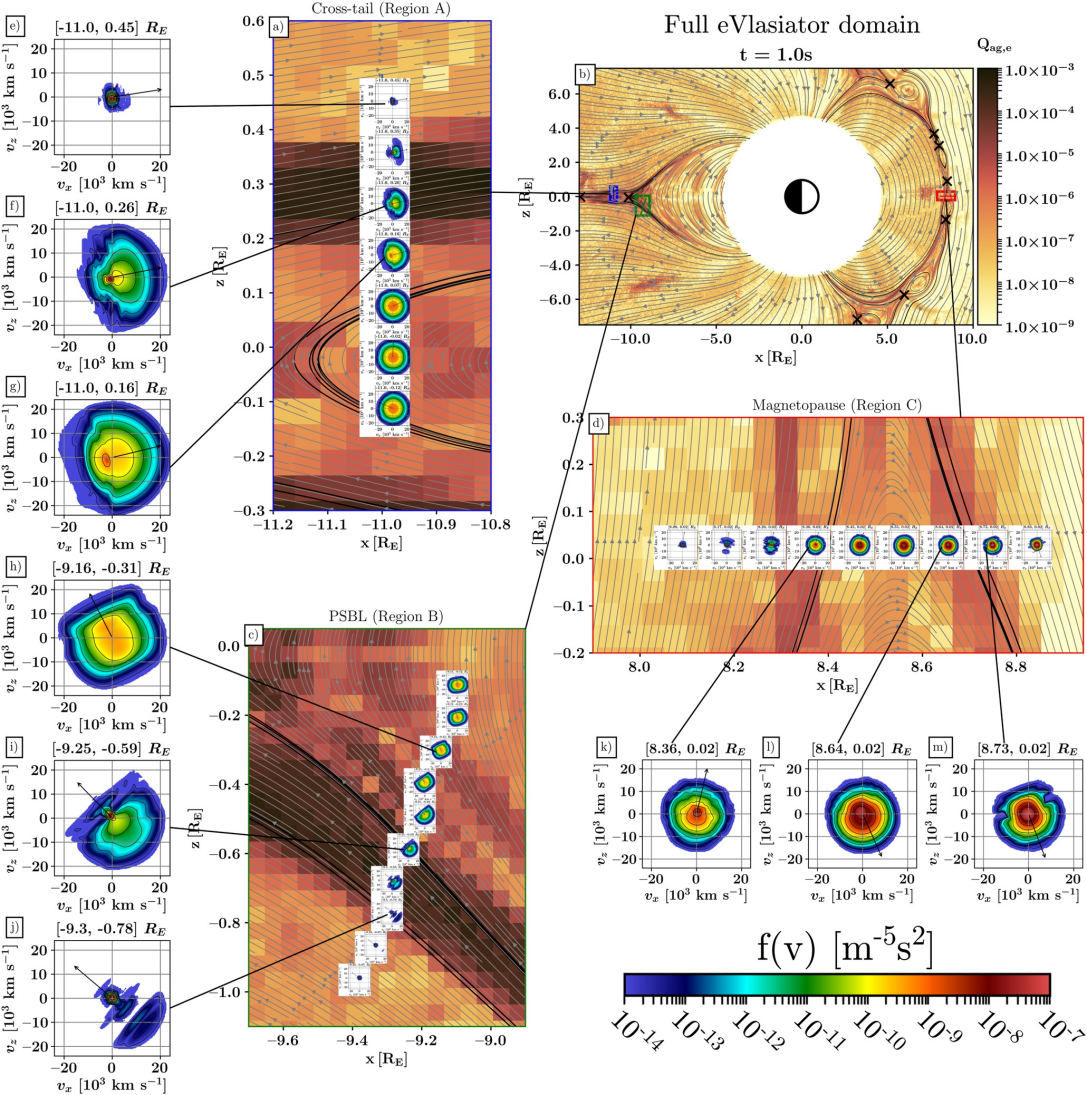
Collections of electron velocity distribution functions (VDFs) of interest as insets in three panels with the top‐right panel showing the location of the smaller panels at the 1s eVlasiator end state. (a) VDFs along a line through the northern plasma sheet boundary layer (PSBL) and the current sheet/magnetic island (Region A). (b) The full simulation domain, with electron agyrotropy as background color, magnetic field lines as gray lines and separatrices as black lines, X‐points as black crosses, and insets (b–d) marked with frames and lines showing lineout positions. (c) VDFs along a crossing from southern lobe through PSBL and into magnetospheric plasma (Region B). (d) VDFs through the dayside magnetopause and reconnection exhaust (Region C). (e–g) Selected VDFs from the tail crossing (Region A). (h–j) Selected VDFs from the nightside boundary crossing (Region B). (k–m) Selected VDFs from the dayside crossing (Region C). The VDF plot range is here ±24,000 km/s in both *v*_*x*_ and *v*_*z*_ (or *v*_∥_ and *v*_⊥_, later on) with the local B vector overlaid as a black arrow.

Given the Vlasiator simulation input (Hoilijoki et al., 2017), eVlasiator produces electron distribution functions describing the response of kinetic electrons to ion‐scale‐driving effects during increased geomagnetic activity. Figure 2 shows an overview of the eVlasiator simulation results with details shown from regions of interest.

As a measure of the departure from Maxwellian populations, we show electron agyrotropy in Figure 2, at certain regions of interest, with electron VDFs embedded along sample virtual spacecraft trajectories (VST) through the snapshot. Agyrotropy is derived from the full electron pressure tensor as described by Swisdak (2016):

(4) $$Q_{\text{ag}}=\frac{P_{12}^{2}+P_{13}^{2}+P_{23}^{2}}{P_{⊥}^{2}+2P_{⊥}P_{∥}},$$

where *P* is the pressure tensor, and *Q*_ag_ ranges from 0 (gyrotropic) to 1 (maximal agyrotropy). Electron agyrotropy evolves during the simulation from initially low values associated with Maxwellian distributions to maximum values of ≈10^−3^, which is similar to MMS observations (Webster et al., 2018). The greatest agyrotropy occurs at boundary layers, such as the magnetopause and the PSBL. We additionally observe enhanced agyrotropy at the X‐lines, indicating electron dynamics driven by ion‐scale magnetic and electric fields despite not resolving electron diffusion regions spatially.

Figure 2b shows the full simulation domain with electron agyrotropy, magnetic field lines, and magnetic separatrices. Overlaid on (b) are the extents of the detail panels (a), (c), and (d) in blue, green, and red, respectively. Panels (a), (c), and (d) show detailed electron VDFs of the simulation along three VSTs, which we will focus our analysis on: Region A: Tail current sheet cross section; Region B: Nightside PSBL crossing; and Region C: Dayside magnetopause crossing. For each of these panels, we further highlight three electron VDFs (e–g), (h–j), and (k–m). The VDFs are shown in the GSE *v*_XZ_ plane, integrated over *v*_y_. The magnetic field direction is overlaid as a black arrow.

**Figure 3:**
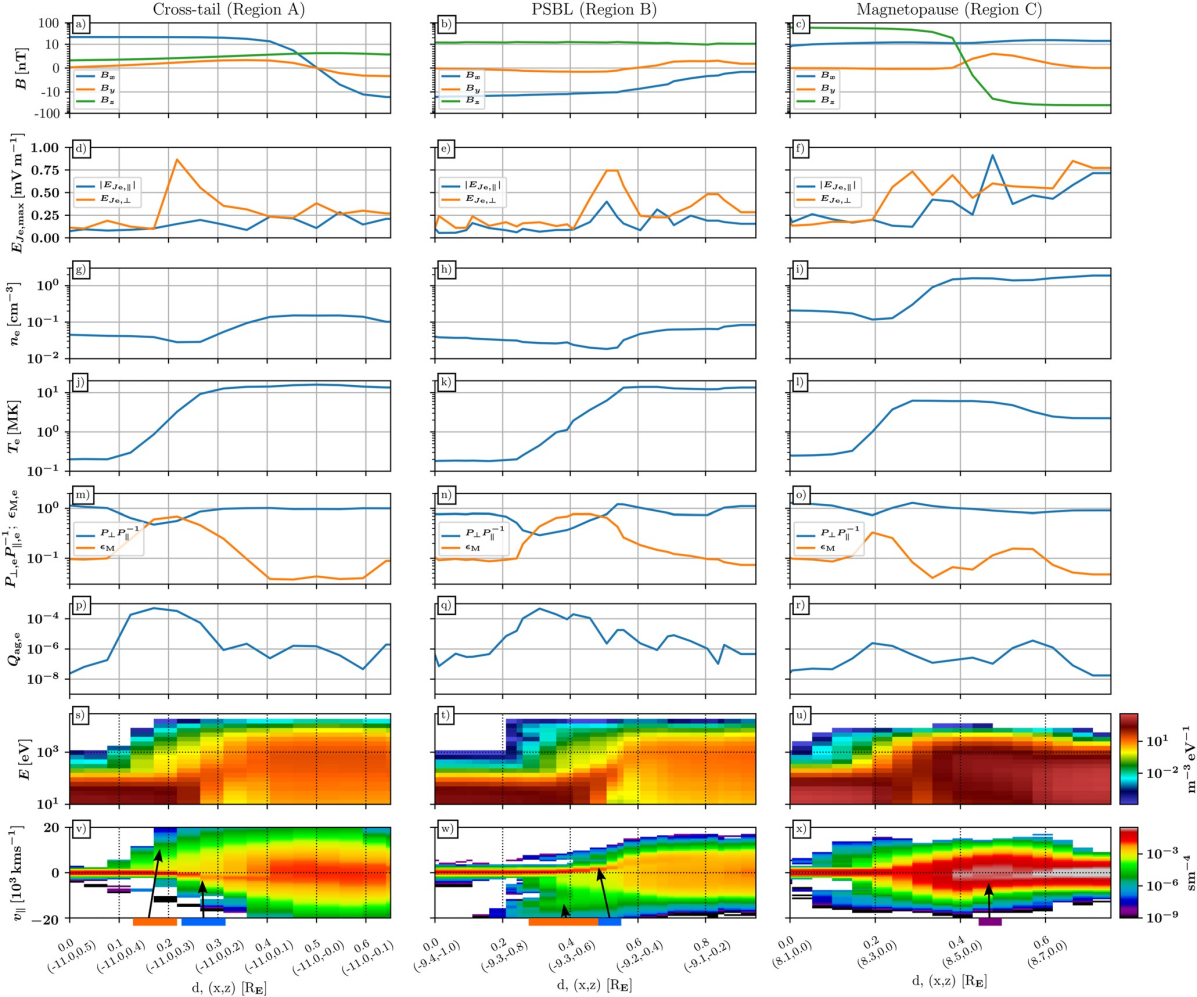
Bulk variables and spectra along the virtual spacecraft trajectories. Columns: Cross‐tail (a), plasma sheet boundary layer (PSBL) (b), and magnetopause (c). Rows: Magnetic field *B*_*x*,*y*,*z*_, electron oscillation electric field $\max\left(\left|E_{J_{e};⊥,∥}\right|\right)$, electron density *n*_e_, electron temperature *T*_e_, electron pressure anisotropy $P_{e,∥}P_{e,⊥}^{-1}$ and non‐Maxwellianity *ϵ*_M_, electron agyrotropy *Q*_ag,e_, energy spectra of the electron velocity distribution functions, and parallel velocity distributions of the electrons. Horizontal axis is given as distances *d* along the virtual spacecraft trajectorie and as a pair of (x and z) coordinates at the tick locations.

In Region A, starting within the northern lobe, we see a cold electron population (e) that when moving toward the current sheet, smoothly transitions to a dense core population through the PSBL (f). There we start to see a hot, parallel beaming component and a parallel velocity for the cool core population in the opposite direction. Notably, f is near a separatrix originating from an active reconnection site tailward of Region A. Deeper in the sheet, the VDFs tend smoothly toward drifting Maxwellian distributions (g; see Figure 3m for explicit nonMaxwellianity measure) with drift speeds similar to ion reconnection outflows ($V_{xz,e^{-}}=\left[732,-62\right]\;km\;s^{-1}$ against $V_{xz,p^{+}}=\left[913,-27\right]\;km\;s^{-1}$ at the middle of the current sheet).

In Region B, starting now from the southern lobe, the cool electron populations again are accompanied by parallel beams along the PSBL (j). Similarly as in Region A, going through the PSBL and separatrices, the core population peak evolves to include low‐energy beaming toward X‐lines along the magnetic field and a hot component is seen beaming in the opposite direction (i). Moving into the dipolar field lines (h), we see the core population become hotter and transition to bi‐parallel beaming.

Lastly, in Region C, we start on the magnetospheric side of the crossing and see the electron population transitions from a cool core population in the magnetosphere to similar inflow‐outflow patterns (k) as in Region B. Crossing the magnetosphere‐side separatrix and the boundary layer, we see the core electron population parallel‐beaming toward the X‐point and a hot exhaust population (l) that is connected to a dayside reconnection point north of the panel (d). Crossing the outbound separatrix, the VDFs tend toward Maxwellian (m) within the magnetosheath.

Figure 3 shows details of bulk quantities and spectra along the three VSTs, mimicking the measurements a satellite traversing the magnetosphere would take. The amplitude of the electron oscillation term (Equation 1) of the electric field, perpendicular $\left(E_{J_{e},⊥}\right)$ and parallel $\left(E_{J_{e},∥}\right)$ to $\vec{B}$, along the VST is obtained as a spatiotemporal maximum: spatially over 5 cells perpendicular to the trajectory and temporally over 5 snapshots at 20 ms intervals preceding and including the 1 s endpoint. We also calculate the explicit non‐Maxwellianity *ϵ*_*M*_ using the method of Graham et al. (2021):

(5) $$\epsilon_{M}=\frac{1}{2n_{e}}\int_{V}|f_{\text{sim}}-f_{\text{model}}|dV,$$

where *f*_sim_ is the VDF given by the simulation and *f*_model_ is a bi‐Maxwellian distribution fitted to the simulation VDF.

For Regions A and B, both crossing the PSBL, we see enhanced $E_{J_{e},⊥}$ oscillations at the PSBL, coincident with the core population shifting to inflows (A: antiparallel, *d* ≈ 0.2–0.3 R_E_, B: parallel *d* ≈ 0.5 R_E_; blue shading on bottom row). In contrast, the regions of hot parallel beaming without core inflow but with enhanced agyrotropy (A: *d* ≈ 0.1–0.2 R_E_, B: *d* ≈ 0.3–0.5 R_E_; orange shading) are not strongly represented in either $E_{J_{e}}$ component. Region C shows strong $E_{J_{e}}$ activity over most of the cut with additionally a strong $E_{J_{e},∥}$ peak visible in the exhaust region (*d* ≈ 0.45 R_E_, purple shading). The coincident electron population exhibits a flattened top in parallel velocity. Magnetosheath regions display nearly as large parallel electric fields as perpendicular, while the magnetosphere side is dominated by the perpendicular component. The inflowing cores (antiparallel on the sheath side, parallel on the magnetosphere side) are not as readily seen in the spectra for Region C.

### 3.2 Detailed Comparison to MMS

Finally, we compare eVlasiator results with in situ previously published observations by MMS, finding corresponding MMS observations for Regions B and C as reported by Nakamura et al. (2016) in the magnetotail and by Khotyaintsev et al. (2020) at the magnetopause, respectively. Region A is included as a reference point for distributions adjacent to (symmetric) reconnection, for which there are other simulation studies (see Discussion). Currently published observations of tail reconnection by Torbert et al. (2018) and Chen et al. (2019, 2020) are deep within EDRs, and we do not reproduce them well with the current grid scales. Future observations may provide more suitable comparisons for Region A.

Khotyaintsev et al. (2020) describe MMS observations of a dayside magnetopause EDR crossing on 2 December 2015, crossing both magnetosheath and magnetosphere side separatrices and the diffusion region itself. They detail electron jets and inflows from dayside reconnection in the electron current sheet close to the magnetosphere separatrix. They also describe electrostatic waves and coincident flat‐top electron distributions in *v*_∥_.

Nakamura et al. (2016) describe MMS observations crossing the southern near‐Earth PSBL during a substorm on 23 June 2015, finding small‐scale field‐aligned currents and corresponding electron distributions. The observations are interpreted to cross the separatrix of an active X‐line with electron inflows and heated outflows observed by the MMS.

**Figure 4:**
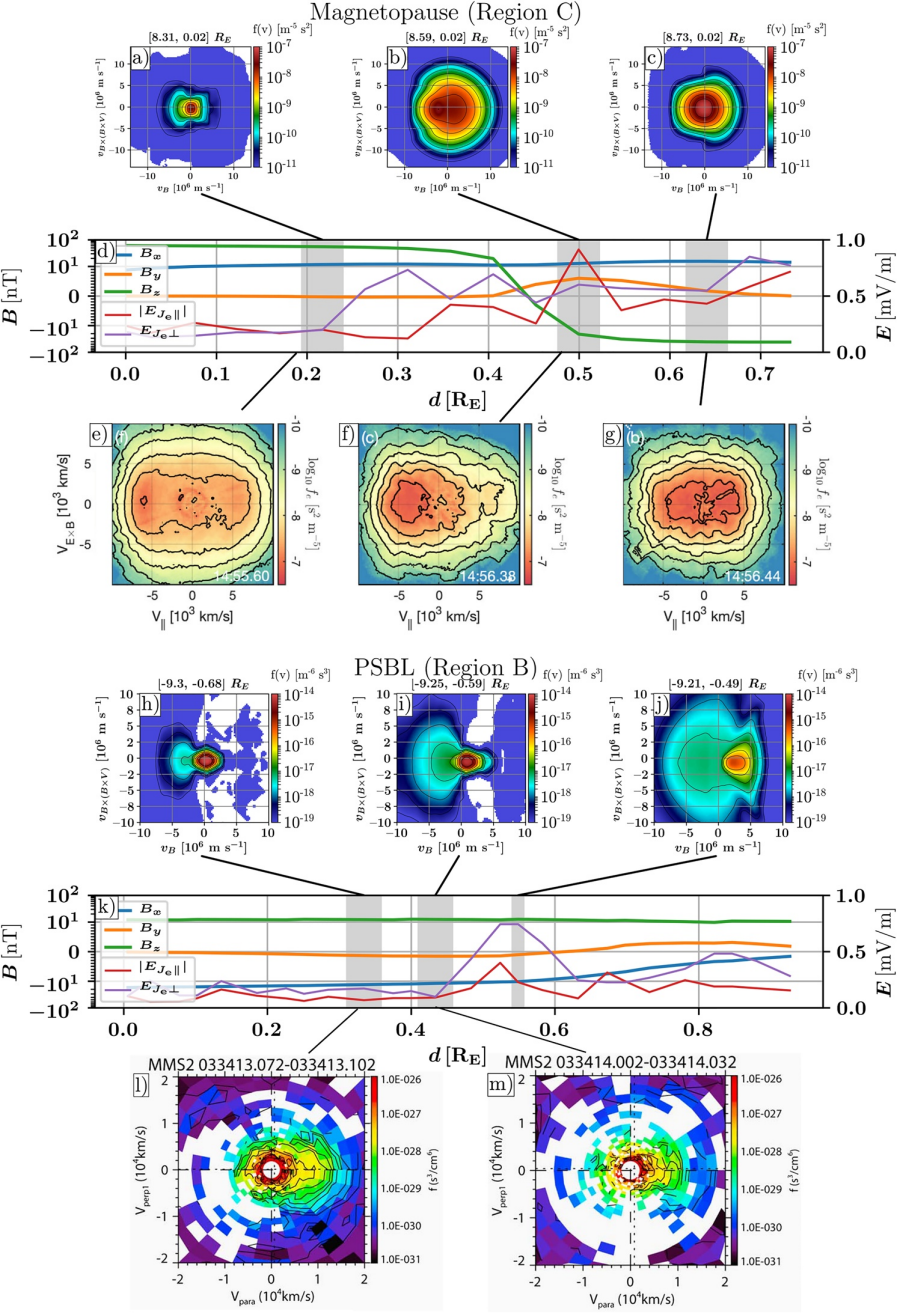
Electron distributions compared to Magnetospheric Multiscale (MMS) observations. Panels (a–g): Magnetopause crossing (Region C) with panels (a–c) showing simulated velocity distribution functions (VDFs), rotated along the magnetic field in the simulation frame. Panel (d) is the magnetic field and $E_{J_{e}}$ as in Figure 3 and (e–g) MMS‐observed VDFs adapted from Khotyaintsev et al. (2020) (Reprinted excerpts of Figure 3 with permission from Khotyaintsev et al., Physical Review Letters, 124, 045101, 2020. http://doi.org/10.1103/PhysRevLett.124.045101. Copyright 2020 by the American Physical Society.) with VDFs (a–c) and (e–g) integrated over plane normal. Panels (h–m): plasma sheet boundary layer (PSBL) crossing (Region B), with panels (h–j) showing virtual VDFs; panel (k) shows the magnetic field and $E_{J_{e}}$ as in Figure 3, and (l and m) are MMS‐observed VDFs adapted from Nakamura et al. (2016) (Reprinted excerpts from Figure 3 under Creative Commons Attribution 3.0 license.) with VDFs (h–j) and (l–m) cut through the plane. The gray‐shaded regions in (d and k) show the extent of the eVlasiator spatial cells of the corresponding VDFs.

Figure 4 shows selected electron distribution functions adjacent to the magnetopause or PSBL reconnection regions from both eVlasiator and MMS. In Region C, on the magnetosphere side, the simulated VDF displays a bi‐parallel configuration (Figure 4a), corresponding to observations (e, Khotyaintsev et al., 2020). Closer to the sheath‐side separatrix (b), we see the core accelerated toward the X‐line, as in observations (f), although a wider, denser distribution presents in the simulation. This coincides with a peak in parallel electric field activity as observed by Khotyaintsev et al. (2020) and in eVlasiator. eVlasiator shows counterstreaming electrons (b) as in observations (f). Panel (c) shows a simulated VDF in the magnetosheath, beginning to show parallel elongation in the core population, which is comparable to the bi‐parallel beaming in observations (g), with continued electric field activity (d).

The comparison between the observed and simulated PSBL crossings (Region B and panels h–m) is somewhat complex due to the dynamic nature of the observed PSBL compared to the static crossing of the PSBL in the simulation. Nonetheless, the overall features of the distributions, including the low‐energy core streaming toward the X‐line seen in observations by Nakamura et al. (2016) (l and m), are present in all simulated VDFs (h‐j: core shifted to a parallel direction). Streaming of the core population increases smoothly as the trajectory goes further into the plasma sheet in the simulation. Bidirectional streaming is seen in (i) and (j), which is reminiscent of observations (m). The localized and transient nature of the observations of Nakamura et al. (2016) contrasts with the steady background and comparatively low spatial resolution of our simulation. The spatial configuration of simulated distributions and the X‐line, with the low‐energy core beam (away from the X‐line) and hot energetic beam streaming from the X‐line (inside the plasma sheet, not visible in MMS VDF plots), is also consistent with the PSBL crossing reported by Varsani et al. (2017), subsequent to the one by Nakamura et al. (2016).

## 4 Summary and Discussion

In this work, we employed the novel eVlasiator method (Battarbee et al., 2021) to model electron distributions in a global magnetosphere simulation, using an ion‐scale, geomagnetically active background from a previous Vlasiator 2D‐3V simulation. We present VDFs at the dayside magnetopause, the tail current sheet, and the PSBL and show that they have a remarkable agreement with MMS observations.

As a point of comparison to other simulations, Hoshino et al. (2001) performed particle‐in‐cell simulations of electrons around tail reconnection and found four distinct types of electron distribution. More recent studies, such as Chen, Hesse, Wang, Bessho, and Daughton (2016); Hesse et al. (2016); and Aunai et al. (2013), display similar features outside of EDRs. We see similar behavior to Hoshino et al. (2001) in the plasma sheet of the global simulation, notably in Region A: cold inflows toward X‐points and hot outflows, somewhat bi‐Maxwellian distributions inside the plasma sheet, gradually transformed to isotropic, near‐Maxwellian distributions at the center of the plasma sheet. This is remarkable as Hoshino et al. (2001) resolve spatially the electron inertial scales (albeit at a lower *m*_e_/*m*_p_), while we do not.

Therefore, these electron dynamics are, at least partially, resolved at larger scales, and reconnection inflows and hot parallel beaming are reproduced with global ion‐scale driving and a subset of kinetic electron physics. Our handling of electron oscillations reproduces parallel electric fields and coincident flat‐top distributions at the magnetopause. This suggests that precise modeling of electron physics at the smallest scales may not be required for practical global models, and ion‐kinetic models can be used as a basis to evaluate the impact of electron dynamics on space weather.

Studies such as the detailed analysis of reconnection regions and magnetosheath turbulence remain the domain of local fully kinetic simulations. Physics and features at electron spatial scales, like electron diffusion regions, are not resolved and may therefore not be reproduced or are under‐resolved. An example of this limitation may be visible in our comparison with the MMS VDFs by Nakamura et al. (2016), who report thin, transient sub‐ion scale current sheets during the studied PSBL crossing. The increased electron mass leads to slower thermalization of the system and slightly modified dispersion relations for the hybrid modes. The coupling between the plasma oscillations and electron Larmor frequency is somewhat underestimated in eVlasiator as *ω*_pe_/*ω*_ce_ is slightly above unity for an unscaled mass ratio in the background simulation and scaled by a factor of $\sqrt{10}$ for the eVlasiator simulation.

## Data Availability

Vlasiator (Pfau‐Kempf et al., 2021, 2022) is distributed under the GPL‐2 open‐source license. The run described here takes several terabytes of disk space and is kept in storage maintained within the CSC‐IT Center for Science. Vlasiator data presented in this paper can be accessed by following the data policy described in https://www2.helsinki.fi/en/researchgroups/vlasiator/rules-of-the-road.
